# Association between Protein Intake and Skeletal Muscle Mass among Community-Dwelling Older Japanese: Results from the DOSANCO Health Study: A Cross-Sectional Study

**DOI:** 10.3390/nu13010187

**Published:** 2021-01-09

**Authors:** Akinori Yaegashi, Takashi Kimura, Takumi Hirata, Shigekazu Ukawa, Koshi Nakamura, Emiko Okada, Takafumi Nakagawa, Akihiro Imae, Akiko Tamakoshi

**Affiliations:** 1 Graduate School of Medicine, Hokkaido University, North 15, West 7, Kita-ku, Sapporo 060-8638, Japan; yaegashi@t.do-bunkyodai.ac.jp (A.Y.); kimura@med.hokudai.ac.jp (T.K.); tamaa@med.hokudai.ac.jp (A.T.); 2 Department of Health and Nutrition, Faculty of Human Science, Hokkaido Bunkyo University, 5-196-1 Kogane-chuo, Eniwa 061-1449, Japan; 3 Graduate School of Human Life Science, Osaka City University, Osaka 558-0022, Japan; ukawa@osaka-cu.ac.jp; 4 Department of Public Health and Hygiene, Graduate School of Medicine, University of the Ryukyus, Okinawa 903-0215, Japan; knakamur@med.u-ryukyu.ac.jp; 5 Department of Nutritional Epidemiology and Shokuiku, National Institute of Biomedical Innovation Health and Nutrition, Tokyo 162-8636, Japan; okadae@nibiohn.go.jp; 6 The Hokkaido Centre for Family Medicine, Sapporo 007-0841, Japan; takafumi.nakagawa@hcfm.jp; 7 Suttsu Municipal Suttsu Clinic, Suttsu 048-0406, Japan; a.imae@hcfm.jp

**Keywords:** epidemiology, muscle mass, older adults, protein intake

## Abstract

Whether the source of dietary protein intake is related to appendicular skeletal muscle mass (AMM) and muscle mass (MM) remains unclear. We conducted this cross-sectional study of 277 residents (115 men, 162 women) aged ≥65 years in Japan to examine the association of the amount of dietary protein intake with AMM and MM. We measured dietary protein intake using a brief self-administered diet history questionnaire. AMM and MM were assessed based on bioelectrical impedance. Multivariable linear regression analyses were used to estimate β coefficients that were adjusted for potential confounders. Among Japanese women aged ≥75 years, but not among women aged 65–74 years, dietary animal protein intake was significantly associated with AMM (β (95% confidence interval (CI)): 0.25 (0.10, 0.40)) and MM (β (95% CI): 0.40 (0.16, 0.64)). However, dietary vegetable protein intake was not associated with AMM (β (95% CI): −0.17 (−0.74, 0.41)) and MM (β (95% CI): −0.30 (−1.23, 0.63)). Furthermore, in men aged ≥65 years, dietary protein intake was not associated with AMM or MM. In conclusion, dietary animal protein intake, but not vegetable protein intake, were positively associated with AMM and MM among this population of Japanese women aged ≥75 years.

## 1. Introduction

Aging involves significant changes in body composition, especially in skeletal muscle mass, which is a major contributor to frailty in older individuals [1]. The depletion of muscle mass predisposes older adults to bone fractures [2] and chronic metabolic diseases, such as type 2 diabetes [2,3] and obesity [2], leading to substantial increases in health care costs [4,5].

Although several underlying mechanisms contribute to age-related reductions in skeletal muscle, inadequate dietary protein intake may accelerate this process [6]. Data from the 2012 National Health and Nutrition Survey in Japan showed that nearly half of the participants did not meet recommended dietary protein levels in terms of prevention of sarcopenia [7]. In addition to consuming inadequate amounts of dietary total protein, older adults may be at risk of consuming inadequate dietary protein of high biological value from animal sources [8]. Dietary animal protein provides more essential amino acids than dietary vegetable protein sources, which can stimulate muscle protein synthesis (MPS) [9,10,11].

According to data from the Health, Aging, and Body Composition study in the United States, dietary total and animal protein intake, but not vegetable protein intake, were associated with an increase in lean mass in older people [12]. Compared with American people, Japanese people consume a lower amount of dietary animal protein (8.9% and 8.8% for men and women in Japan, and 10.2% and 10.1% in America) [13]. In addition, because of the difference in food intake between the residents of these countries, the proportions of amino acids consumed by them also differs [13,14].

Therefore, among the Japanese older population, it remains unclear whether total dietary protein and/or the source of dietary protein intake (i.e., animal or vegetable) is associated with appendicular skeletal muscle mass (AMM) and muscle mass (MM). These questions are of importance because Japan is a leading aging society. Thus, the aim of this study was to assess the association of the amount and source of dietary protein with AMM and MM among community-dwelling older adults living in Japan. This study may expand the new aspect of understanding a relationship between the amount and source of dietary protein with AMM and MM and also be useful for specific nutritional strategies concerning protein intakes among community-dwelling older Japanese.

## 2. Materials and Methods 

### 2.1. Study Design and Population

A cross-sectional study was conducted as part of the Dynamics of Lifestyle and Neighborhood Community on Health Study (DOSANCO Health Study), a community-based survey conducted in the town of Suttu, Hokkaido, Japan, between May and November 2015. All participants agreed to participate voluntarily.

The target population for the DOSANCO Health Study were all residents aged three years and older who lived in the town of Suttu, Hokkaido, Japan. Of these, we excluded participants living in nursing homes. The survey consisted of two parts: a questionnaire survey and an anthropometric survey. The questionnaire survey consisted of a series of items on lifestyle, health, mental health, community, and eating habits. The anthropometric survey was conducted among participants who responded to the questionnaire and included blood sampling and measurements of height, weight, physical activity, and body composition. The study protocol was carried out following the rules of the Declaration of Helsinki of 1975, revised in 2013, and was approved by the Institutional Review Committee for Ethical Issues of the Faculty of Medicine, Hokkaido University (15-002). Written informed consent was obtained from all participants.

### 2.2. Assessment for Dietary Intake

Dietary protein and energy intake during the previous month were assessed using a brief self-administered diet history questionnaire (BDHQ). Details of the BDHQ have been described elsewhere [15,16]. Briefly, the BDHQ used in this study is a 10-page fixed-portion questionnaire used to estimate the dietary intake of 58 food items. The food items and portion sizes comprising the BDHQ were derived primarily from a food list used in the National Health and Nutrition Survey, Japan, and from several Japanese recipe books [17]. Dietary protein and energy intakes were estimated depending on the frequency of consumption of each food and beverage, assumed portion size, and protein and energy contents derived from the Standard Tables of Food Composition in Japan using an ad hoc computer algorithm for the BDHQ [17]. Dietary protein from fish, shellfish, meat, eggs, and dairy products were considered to be animal sources of protein. Dietary protein from cereals, pulses, potatoes, fruits, vegetables, confectionaries, alcoholic beverages, and non-alcoholic beverages were considered to be vegetable sources of protein. The proportion of each food group’s contribution to the total dietary protein was calculated by dividing the daily protein of each food group by the total individual daily protein. Pearson’s correlation coefficients of dietary total protein intake between the 16 d dietary record and the BDHQ in 92 men aged 32–76 was 0.38, and 0.35 in 92 women aged 31–69 [16]. Finally, the correlation between a three-day non-consecutive dietary record and the BDHQ in 36 men and 44 women aged 82–94 was 0.32 [18]. Dietary protein intake values were energy-adjusted by a density method (% energy) for the analysis to minimize the influence of dietary misreporting [19]. Intake from supplements was not included in the analysis.

### 2.3. Measurement for Anthropometry and Body Composition

The height of the participants was measured using a calibrated scale. Body weight (in kg, to the nearest 0.1 kg) and body composition by bioelectrical impedance analysis (BIA) were assessed using InBody 430 (Inbody Japan Corporation, Tokyo, Japan) after overnight fasting. As participants wore light clothes and no shoes, weight was corrected by subtracting 1 kg. AMM (kg) and MM (kg) were obtained from the BIA. AMM was divided by height squared to obtain the appendicular skeletal muscle mass index (AMMI; kg/m^2^). Body mass index (BMI) was calculated as weight (kg)/height^2^ (m^2^).

### 2.4. Measurement of Other Variables

Age, sex, physical activity, and current smoking were collected using a self-administered questionnaire. Physical activity was calculated based on the Global Physical Activity Questionnaire (GPAQ). The GPAQ [20] was developed by the World Health Organization for physical activity surveillance across countries. It collects information on physical activity participation in three settings (or domains) as well as sedentary behavior through 16 questions (P1-P16). The domains are “activity at work,” “travel to and from places,” and “recreational activities” [21]. Metabolic equivalent (MET)-minutes/week scores were calculated separately for individual domains and sub-domains, adopting the existing guidelines of the GPAQ, and summed to obtain MET-minutes/week scores for all domains. MET-minutes/week scores were classified into two groups as follows: inactive/low (<600 MET-min/week) and active (≥600 MET-min/week). 

### 2.5. Statistical Analysis

Participants characteristics (continuous variables) were expressed as mean ± standard deviation (SD), and categorical variables were presented as percentages. All analyses were performed separately by sex. Differences across tertile of energy-adjusted total protein intake were examined by chi square test for categorical variables and by analysis of variance (ANOVA) for continuous variables. To examine the association of dietary protein with AMMI, AMM, and MM, multivariable linear regression analysis was used to calculate the coefficients after adjustment for age (years, as a continuous variable), physical activity (MET-minutes/week, <600 or ≥600 or missing), and current smoking (yes or no). We checked the homoscedasticity and the normality of the residuals using the residual plot, and the data was fairly homoscedastic and the residuals were almost normally distributed. Protein intake × age, physical activity, and current smoking interactions were tested, and age was significant. In general, Japanese people aged ≥65 years are classified into two groups: the pre-old age (65–74 years) and the old age (≥75 years) [22], because most Japanese people aged 65–74 years maintained mental and physical health well. We considered that the association between dietary protein intake and AMM or MM differed by age, and age-stratified analyses was performed in the present study. All statistical analyses were performed using the SAS statistical software package (version 9.4 for Windows; SAS Institute, Cary, NC, USA). All probability values were two-tailed, and the significance level was set at *p* < 0.05. 

## 3. Results

**Figure 1:**
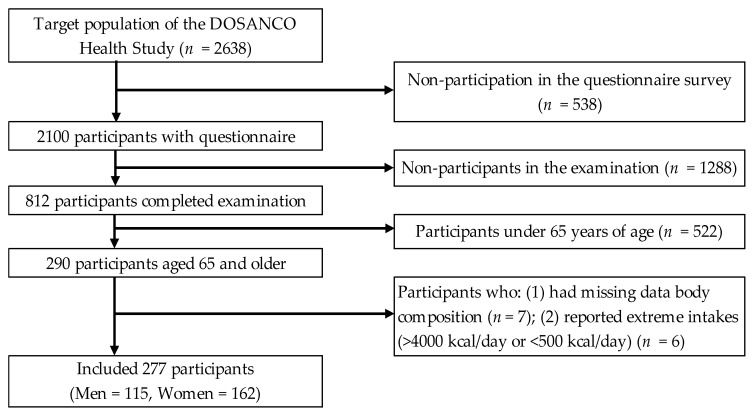
Flow diagram of the study participants.

A total of 2100 participants (977 men and 1123 women) completed the questionnaire survey (response rate: 79.6%). Of these, 812 subjects participated in the anthropometric survey, and 290 participants aged ≥ 65 years were eligible for the present study. We excluded 13 participants for the following reasons: (1) missing data on body composition (*n* = 7) and (2) extreme energy intakes (>4000 kcal/day or <500 kcal/day; *n* = 6). Finally, we included 277 patients (115 men and 162 women) for the analysis in the present study. An overview of the study procedures is presented in Figure 1.

In men, the mean (± SD) age of the participants was 74.5 ± 7.0 years. The mean intake of dietary protein was 76.6 ± 35.7 g/day and 16.0 ± 3.4% energy. Mean AMMI, AMM, and MM were 9.6 ± 1.0 kg/m^2^, 25.7 ± 3.3 kg, and 44.4 ± 5.2 kg, respectively. In women, the mean (± SD) age of the participants was 75.2 ± 7.0 years. The mean intake of dietary protein was 69.5 ± 31.5 g/day and 16.7 ± 3.5% energy. Mean AMMI, AMM, and MM were 8.2 ± 0.8 kg/m^2^, 18.2 ± 2.4 kg, and 32.6 ± 3.8 kg, respectively. The characteristics of the study population by tertile (T) of energy-adjusted total protein intake are shown in Table 1.

The associations between dietary protein intake and AMMI, AMM, and MM in participants aged ≥65 years are shown in Table 2. In women, dietary total protein and animal protein intake were significantly associated with AMM (β (95% confidence interval (CI)): 0.10 (0.01, 0.20) and 0.10 (0.01, 0.19)) and MM (β (95% CI): 0.17 (0.01, 0.33) and 0.17 (0.02, 0.32)) after adjusting for age, physical activity, and current smoking. However, vegetable protein intake was not associated with AMM (β (95% CI): −0.11 (−0.43, 0.21) or MM (β (95% CI): −0.19 (−0.71, 0.34)) after adjusting for age, physical activity, and current smoking. 

The associations between dietary protein intake and AMMI, AMM, and MM in participants by age-stratified (65–74, ≥75) are shown in Table 3. In women aged ≥75, dietary total protein and animal protein intake were significantly associated with AMMI (β (95% CI): 0.07 (0.02, 0.13) and 0.07 (0.02, 0.12)), AMM (β (95% CI): 0.26 (0.11, 0.42) and 0.25 (0.10, 0.40)), and MM (β (95% CI): 0.42 (0.17, 0.67) and 0.40 (0.16, 0.64)) after adjusting for age, physical activity, and current smoking. However, in women aged 65–74, dietary total protein and animal protein intake were not associated with AMMI (β (95% CI): −0.02 (−0.06, 0.03) and −0.02 (−0.06, 0.02)), AMM (β (95% CI): −0.02 (−0.14, 0.10) and −0.01 (−0.12, 0.11)), and MM (β (95% CI): −0.03 (−0.23, 0.16) and −0.01 (−0.19, 0.17)) after adjusting for age, physical activity, and current smoking. 

In men, dietary protein intake was not associated with AMMI, AMM, and MM.

## 4. Discussion

We found that dietary total protein and animal protein intake were associated with AMM and MM among older Japanese women aged ≥65 years, but vegetable protein intake was not associated with AMM or MM. In addition, in men aged ≥65 years, total dietary protein and the source of dietary protein intake were not associated with AMM or MM. 

Evidence from experimental research indicates that the potential of protein to stimulate MPS is mainly determined by its leucine content, which is high in animal proteins [23]. Additionally, most animal proteins are considered to be of higher quality than vegetable proteins in terms of amino acid composition, digestibility, and bioavailability [23]. Our results showed that dietary animal protein intake, but not total or vegetable protein intake, was associated with AMM and MM, which is in line with the findings of previous experimental studies [23].

The results of an epidemiological study in America examining the association between dietary animal protein intake and AMM are consistent with ours [12]. Houston et al. indicated that dietary animal protein intake, but not vegetable protein intake, was positively associated with changes in lean mass over a period of three years among community-dwelling older adults (70–79 years) [12]. Thus, the results of their study and ours show that animal protein is important for the maintenance of AMM and MM not only in America, but also in Japan. However, the range of intake was much wider for animal protein than for vegetable protein, which may have limited our ability to detect a significant association of dietary vegetable protein intake with AMM and MM. 

We showed that, in the present study, dietary total protein and animal protein intake were positively associated with AMM and MM among women, but not in men. This might be caused by the higher MPS of older women compared with that of older men reported in some studies [24,25]. One study in healthy individuals observed that the muscle protein fractional synthesis rate as well as whole-body protein synthesis were higher in women than in men in old age [25]. In addition, the observational study by Elstgeest et al. [26] was consistent with ours, and they did not observe significant associations between dietary protein intake and change in AMM over three years in men; however, among women, a higher protein intake at baseline was associated with less loss of AMM over three years.

Meanwhile, we showed dietary total protein and animal protein intake was positively associated with AMMI, AMM, and MM among women aged ≥75, not aged 65–74. This is consistent with the results of previous experimental studies which examined the relationship between dietary protein intake and hand grip strength in Japan [27]. This result imply that dietary intervention alone might be a less effective strategy to prevent age-related loss of AMM and MM for the women aged 65–74. For their population, strategies other than dietary interventions to maintain AMM and MM might be appropriate, such as exercise.

Several limitations warrant mention. First, the present study was conducted under a cross-sectional design, which prevents the investigation of the causal effect of protein intake on AMM and MM. Second, we were unable to include the intake of dietary supplements in the calculation of protein intake. However, the use of supplements containing mainly protein or amino acids is uncommon in Japanese adult men (1.1%) and women (1.5%) [28]. Therefore, any influence of supplements on protein intake is probably low. 

## 5. Conclusions

We found that dietary total protein and animal protein intake were positively associated with AMM and MM among Japanese women aged ≥75 years, not among Japanese women aged 65–74 years. While we cannot establish a causal association, these results suggest that low dietary protein intake, especially dietary animal intake, may be a modifiable risk factor for loss of AMM and MM in older Japanese women aged ≥75 years.

## Figures and Tables

**Table 1:** Characteristics of study participants from Suttu town, Hokkaido, Japan, by tertile (T) of energy-adjusted total protein intake.

	Men										Women									
	T1			T2			T3			*p*	T1			T2			T3			*p*
Age, y	73.4	±	7.5	74.0	±	6.9	76.1	±	6.6	0.20	75.8	±	7.6	74.4	±	6.8	75.5	±	6.7	0.59
BMI, kg/m^2^	24.3	±	3.2	24.0	±	3.3	23.5	±	3.1	0.58	23.0	±	3.4	24.0	±	3.5	24.1	±	3.5	0.19
AMMI, kg/m^2^	9.9	±	0.8	9.5	±	1.0	9.4	±	1.0	0.04	8.0	±	0.9	8.4	±	0.7	8.2	±	0.7	0.03
AMM, kg	26.5	±	2.9	25.5	±	3.6	25.1	±	3.4	0.17	17.7	±	2.6	18.6	±	2.3	18.4	±	2.1	0.10
MM, kg	45.6	±	4.6	44.1	±	5.6	43.6	±	5.4	0.20	31.7	±	4.1	33.3	±	3.7	32.9	±	3.4	0.08
GPAQ, *n* (%)																				
active (≥600 MET-mins/week)	24 (63.1)			24 (61.5)			19 (50)			0.40	26 (48.2)			28 (51.9)			29 (53.7)			0.93
inactive (<600 MET-mins/week)	14 (36.8)			14 (35.9)			19 (50)				26 (48.2)			26 (48.2)			25 (46.3)			
missing	0 (0)			1 (2.6)			0 (0)				2 (3.7)			0 (0)			0 (0)			
Current smoking, *n* (%)	8 (21.1)			11 (28.2)			5 (13.2)			0.27	6 (11.2)			1 (1.9)			3 (5.6)			0.13
Total energy intake, kcal/d	1883	±	514	1802	±	577	2005	±	745	0.36	1497	±	461	1657	±	534	1781	±	702	0.04
Total protein intake, % energy	12.6	±	1.5	15.7	±	0.7	19.8	±	2.6	<0.001	13.0	±	1.4	16.5	±	0.9	20.5	±	2.5	<0.001
Animal protein intake, % energy	6.5	±	1.6	9.2	±	1.3	13.3	±	3.3	<0.001	6.2	±	1.6	9.5	±	1.3	13.7	±	3.1	<0.001
Vegetable protein intake, % energy	6.1	±	1.2	6.5	±	0.9	6.5	±	1.4	0.22	6.8	±	0.9	7.0	±	1.0	6.8	±	1.3	0.41
Fat intake, % energy	21.6	±	4.5	25.9	±	4.9	29.4	±	4.7	<0.001	22.5	±	5.6	27.9	±	4.7	31.6	±	4.3	<0.001
Carbohydrate intake, % energy	57.5	±	8.4	53.6	±	7.5	47.1	±	6.7	<0.001	61.6	±	7.5	54.2	±	4.5	46.7	±	6.0	<0.001
Total protein intake, g/d	59.2	±	18.0	70.5	±	22.2	100.4	±	46.4	<0.001	48.8	±	16.2	68.4	±	22.9	91.2	±	36.1	<0.001
Animal protein intake, g/d	30.9	±	12.3	40.7	±	12.4	68.3	±	38.7	<0.001	23.4	±	9.9	39.6	±	15.1	61.7	±	28.3	<0.001
Vegetable protein intake, g/d	28.3	±	8.3	29.8	±	12.1	32.1	±	12.1	0.32	25.3	±	8.0	28.9	±	9.5	29.5	±	10.5	0.04
Fat intake, g/d	44.6	±	13.7	52.1	±	19.5	65.4	±	27.0	<0.001	38.3	±	16.0	51.2	±	18.7	63.2	±	27.5	<0.001
Carbohydrate intake, g/d	270.0	±	81.8	240.3	±	79.9	234.1	±	88.3	0.14	229.0	±	73.2	224.8	±	75.3	206.6	±	85.5	0.29

BMI, body mass index; AMMI, appendicular skeletal muscle mass index; AMM, appendicular skeletal muscle mass; MM, muscle mass; GPAQ, Global Physical Activity Questionnaire; MET, Metabolic equivalent; analysis of variance (ANOVA) or chi-square tests were used to evaluate the distribution; Mean ± SD or *n* (%).

**Table 2:** Associations of dietary protein with appendicular skeletal muscle mass index, appendicular skeletal muscle mass, and muscle mass by sex.

	AMMI	AMM	MM
	β (95% CI) (SE)	β (95% CI) (SE)	β (95% CI) (SE)
Men (*n* = 115)			
Total protein intake, % energy			
age adjusted	−0.04 (−0.09, 0.01) (0.02)	−0.04 (−0.21, 0.12) (0.08)	−0.06 (−0.33, 0.21) (0.13)
Multiple adjusted model	−0.02 (−0.07, 0.02) (0.02)	0.01 (−0.16, 0.17) (0.08)	0.02 (−0.24, 0.29) (0.13)
Animal protein intake, % energy			
age adjusted	−0.04 (−0.09, 0.004) (0.02)	−0.07 (−0.22, 0.09) (0.08)	−0.09 (−0.34, 0.17) (0.13)
Multiple adjusted model	−0.02 (−0.07, 0.02) (0.02)	−0.03(−0.18, 0.13) (0.08)	−0.02 (−0.28, 0.23) (0.13)
Vegetable protein intake, % energy			
age adjusted	0.04 (−0.09, 0.18) (0.07)	0.22 (−0.25, 0.70) (0.24)	0.30 (−0.45, 1.06) (0.38)
Multiple adjusted model	0.05 (−0.09, 0.18) (0.07)	0.26 (−0.20, 0.72) (0.23)	0.36 (−0.37, 1.09) (0.37)
Women (*n* = 162)			
Total protein intake, % energy			
age adjusted	0.03 (−0.01, 0.06) (0.02)	0.12 (0.02, 0.21) (0.05)	0.19 (0.04, 0.35) (0.08)
Multiple adjusted model	0.02 (−0.01, 0.06) (0.02)	0.10 (0.01, 0.20) (0.05)	0.17 (0.01, 0.33) (0.08)
Animal protein intake, % energy			
age adjusted	0.02 (−0.01, 0.05) (0.02)	0.11 (0.02, 0.21) (0.05)	0.19 (0.04, 0.33) (0.07)
Multiple adjusted model	0.02 (−0.01, 0.05) (0.02)	0.10 (0.01, 0.19) (0.05)	0.17 (0.02, 0.32) (0.07)
Vegetable protein intake, % energy			
age adjusted	0.03 (−0.08, 0.15) (0.06)	−0.12 (−0.44, 0.20) (0.16)	−0.20 (−0.71, 0.32) (0.26)
Multiple adjusted model	0.02 (−0.09, 0.14) (0.06)	−0.11 (−0.43, 0.21) (0.16)	−0.19 (−0.71, 0.34) (0.26)

Multiple adjusted model included age (continuous), physical activity (categorical; <600 or ≥600 MET-mins/week), and current smoking (yes or no); SE, standard error; AMMI, appendicular skeletal muscle mass index; AMM, appendicular skeletal muscle mass; MM, muscle mass; CI, confidence interval; β, partial regression coefficient; Regression coefficients represent the change in AMMI, AMM, and MM per 1% energy increase of protein intake.

**Table 3:** Associations of dietary protein with appendicular skeletal muscle mass index, appendicular skeletal muscle mass, and muscle mass by age (65–74 or ≥75).

	AMMI	AMM	MM
	β (95% CI) (SE)	β (95% CI) (SE)	β (95% CI) (SE)
Men			
Age (65–74) (*n* = 64)			
Total protein intake, % energy			
Multiple adjusted model	0.02 (−0.05, 0.08) (0.03)	0.12 (−0.11, 0.35) (0.12)	0.16 (−0.21, 0.54) (0.19)
Animal protein intake, % energy			
Multiple adjusted model	−0.003 (−0.07, 0.06) (0.03)	0.04 (−0.19, 0.27) (0.12)	0.04 (−0.33, 0.41) (0.18)
Vegetable protein intake, % energy			
Multiple adjusted model	0.12 (−0.03, 0.27) (0.08)	0.44 (−0.10, 0.99) (0.27)	0.69 (−0.18, 1.55) (0.43)
Age (≥75) (*n* = 51)			
Total protein intake, % energy			
Multiple adjusted model	−0.05 (−0.13, 0.03) (0.04)	−0.03 (−0.29, 0.22) (0.13)	−0.01 (−0.42, 0.39) (0.20)
Animal protein intake, % energy			
Multiple adjusted model	−0.04 (−0.11, 0.03) (0.04)	−0.03 (−0.26, 0.21) (0.12)	0.002 (−0.37, 0.38) (0.19)
Vegetable protein intake, % energy			
Multiple adjusted model	−0.06 (−0.30, 0.19) (0.12)	−0.02 (−0.80, 0.77) (0.39)	−0.15 (−1.40, 1.11) (0.62)
Women			
Age (65–74) (*n* = 86)			
Total protein intake, % energy			
Multiple adjusted model	−0.02 (−0.06, 0.03) (0.02)	−0.02 (−0.14, 0.10) (0.06)	−0.03 (−0.23, 0.16) (0.10)
Animal protein intake, % energy			
Multiple adjusted model	−0.02 (−0.06, 0.02) (0.02)	−0.01 (−0.12, 0.11) (0.06)	−0.01 (−0.19, 0.17) (0.09)
Vegetable protein intake, % energy			
Multiple adjusted model	0.05 (−0.10, 0.19) (0.07)	−0.14 (−0.51, 0.23) (0.18)	−0.22 (−0.81, 0.38) (0.30)
Age (≥75) (*n* = 76)			
Total protein intake, % energy			
Multiple adjusted model	0.07 (0.02, 0.13) (0.03)	0.26 (0.11, 0.42) (0.08)	0.42 (0.17, 0.67) (0.12)
Animal protein intake, % energy			
Multiple adjusted model	0.07 (0.02, 0.12) (0.03)	0.25 (0.10, 0.40) (0.07)	0.40 (0.16, 0.64) (0.12)
Vegetable protein intake, % energy			
Multiple adjusted model	−0.04 (−0.23, 0.16) (0.10)	−0.17 (−0.74, 0.41) (0.29)	−0.30 (−1.23, 0.63) (0.47)

Multiple adjusted model included age (continuous), physical activity (categorical; <600 or ≥600 MET-mins/week), and current smoking (yes or no); SE, standard error; AMMI, appendicular skeletal muscle mass index; AMM, appendicular skeletal muscle mass; MM, muscle mass; CI, confidence interval; β, partial regression coefficient; regression coefficients represent the change in AMMI, AMM, and MM per 1% energy increase of protein intake.

## Data Availability

The data presented in the present study are not publicly available due to privacy and ethical restrictions.
